# Τhe complete mitochondrial genomes of *Ceratitis rosa* and *Ceratitis quilicii*, members of the *Ceratitis* FAR species complex (Diptera: Tephritidae)

**DOI:** 10.1080/23802359.2021.1899073

**Published:** 2021-03-18

**Authors:** Elena Drosopoulou, Aristi Damaskou, Angeliki Markou, Sunday Ekesi, Fathiya Khamis, Aruna Manrakhan, Antonios A. Augustinos, George Tsiamis, Kostas Bourtzis

**Affiliations:** a Faculty of Sciences, Department of Genetics, Development and Molecular Biology, School of Biology, Aristotle University of Thessaloniki, Thessaloniki, Greece; b International Centre of Insect Physiology and Ecology, Nairobi, Kenya; c Citrus Research International, Nelspruit, South Africa; d Insect Pest Control Laboratory, Joint FAO/IAEA Programme of Nuclear Techniques in Food and Agriculture, Seibersdorf, Austria; e Laboratory of Systems Microbiology and Applied Genomics, Department of Environmental Engineering, University of Patras, Agrinio, Greece

**Keywords:** Mitogenome, Natal fruit fly, phylogenetic relations, species delimitation, sterile insect technique

## Abstract

*Ceratitis* FAR is an African species complex comprising insect pests of great economic interest and obscure species limits. Here, we report the mitochondrial genomes of two members of the FAR complex, namely *Ceratitis rosa* and the recently characterized *Ceratitis quilicii*. A phylogenetic analysis based on PCGs of available Tephritidae mitogenomes is presented. The current mitochondrial sequences from the FAR complex could contribute toward the resolution of phylogenetic relationships and species limits within this taxonomically challenging group, which is also an important issue for the development of environment-friendly and species-specific control methods, such as the sterile insect technique (SIT).

*Ceratitis rosa* (Karsch, 1887), the Natal fruit fly, and *Ceratitis quilicii* (De Meyer, Mwatawala & Virgilio sp. Nov) are African insect pests and members of the *Ceratitis* FAR species complex (Diptera: Tephritidae) which also includes *Ceratitis fasciventris* and *Ceratitis anonae*. *Ceratitis quilicii* has been recently described as a different species from *C. rosa* based on molecular genetics, morphometrics, developmental physiology, behavior and sexual compatibility, chemo ecology, and environmental preferences data (reviewed in De Meyer et al. 2016). The two species are almost identical (only adult males exhibit minor differences in the mid tibia) and have overlapping distribution in eastern and southern Africa (De Meyer 2001; De Meyer et al. 2016; Copeland et al. 2006). They are both highly destructive as they attack a great number of wild and cultivated plants causing great economic consequences (De Meyer 2001; De Meyer et al. 2002, 2016; Copeland et al. 2006). Furthermore, there is concern about their invasive potential, since *C. rosa* has been reported in the islands of Mauritius and La Réunion (White et al. 2001). Later it was found that the La Reunion populations of *C. rosa* belong to the group R2 which was subsequently described as *C. quilicii* (Virgilio et al. 2013; De Meyer et al. 2016). In this study we present the first complete mitochondrial genomes of *C. rosa* and *C. quilicii,* which could be useful for further analyses aiming to ascertain the species number and clarify the evolutionary relationships within the FAR complex.

The analyzed specimens came from *C. rosa* and *C. quilicii* laboratory colonies held at the Insect Pest Control Laboratory (IPCL, Seibersdorf, Vienna). The original *C. rosa* colony was established at the International Center of Insect Physiology and Ecology (ICIPE, Kenya) from insects from guava fruits collected from Kibarani, Msambweni district (S 04°19′62.8′′; E 039°32′41.1′′; 34 masl). The original *C. quilicii* colony was established at the Citrus Research International (CRI, South Africa) from samples of infested Jambos and loquat collected from Pretoria (S 25°45′13.7′′; E 28°13′45′′). Both colonies have been verified by experts and used in previous studies (Tanga et al. 2015, 2018). Total genomic DNA was extracted from the whole body of individual adult flies using the DNeasy Blood and Tissue kit (Qiagen). DNA from specimens of the above colonies are kept in IPCL. Library construction and sequencing were performed by Macrogen using pair-ended (2 × 250bp) HiSeq 2500 technology.

Mitogenomes were assembled from the quality-trimmed sequencing reads using the mitochondrial baiting and iterative mapping algorithm MITObim (Hahn et al. 2013) with default parameters. The mitogenome of *C. fasciventris* (NC_035497) was used as the initial template. Annotation was performed using the MITOS WebServer (Bernt et al., 2013) followed by manual curation using BLASTn (Altschul et al. 1997) and Clustal Omega (Sievers et al. 2011) alignments to the mitogenomes of *C. capitata* (NC_000857) and *C. fasciventris* (NC_035497). For the confirmation of tRNA annotations, the tRNAscan-SE (Lowe and Eddy 1997) was used. Maximum-likelihood analysis based on the 13 PCGs from Tephritidae species was inferred in W-IQ-TREE (Trifinopoulos et al. 2016) using the General Time Reversible model with empirical base frequencies and invariable site plus discrete Gamma mode (GTR + F + I + G4) and ultrafast bootstrap analysis (1000 replicates).

The length of the *C. rosa* and *C. quilicii* mitogenomes was 16,047 and 16,035 bp and the A + T content 77.4 and 77.5%, respectively. Each mitogenome contains 13 PCGs (*ND1-6, ND4L, COI-III, ATP8, ATP6,* and *CYTB*) two rRNA (*12S* and *16S rRNA*) and 22 tRNA genes, and one major non-coding sequence, the control region (CR), following the typical organization of insect mitogenome (Cameron 2014). Six PCGs start with ATG, five with ATT, one with ATA and one with TCG, while ten terminate with TAA, one with TAG, and two with an incomplete stop-codon (T). The length of the CR is 1028 bp for *C. rosa* and 1036 bp for *C. quilicii*. The longest intergenic spacer lies between *tRNA^Ile^* and *tRNA^Gln^* for both species, similarly to *C. fasciventris* (Drosopoulou et al. 2017). The two analyzed mitogenomes present 97.75% sequence identity to each other and a very high identity to the mitogenome of *C. fasciventris* (97.85% for *C. rosa* and 98.32% for *C. quilicii*). The phylogenetic analysis among available Tephritidae mitogenomes clusters the currently analyzed species in a well-supported clade (Figure 1), confirming their close phylogenetic relationships. The availability of additional FAR mitogenome sequences in the future is expected to provide useful information for the better understanding of species identities within the complex, which is necessary for the development and application of environment-friendly control methods, such as the SIT.

**Figure 1.**
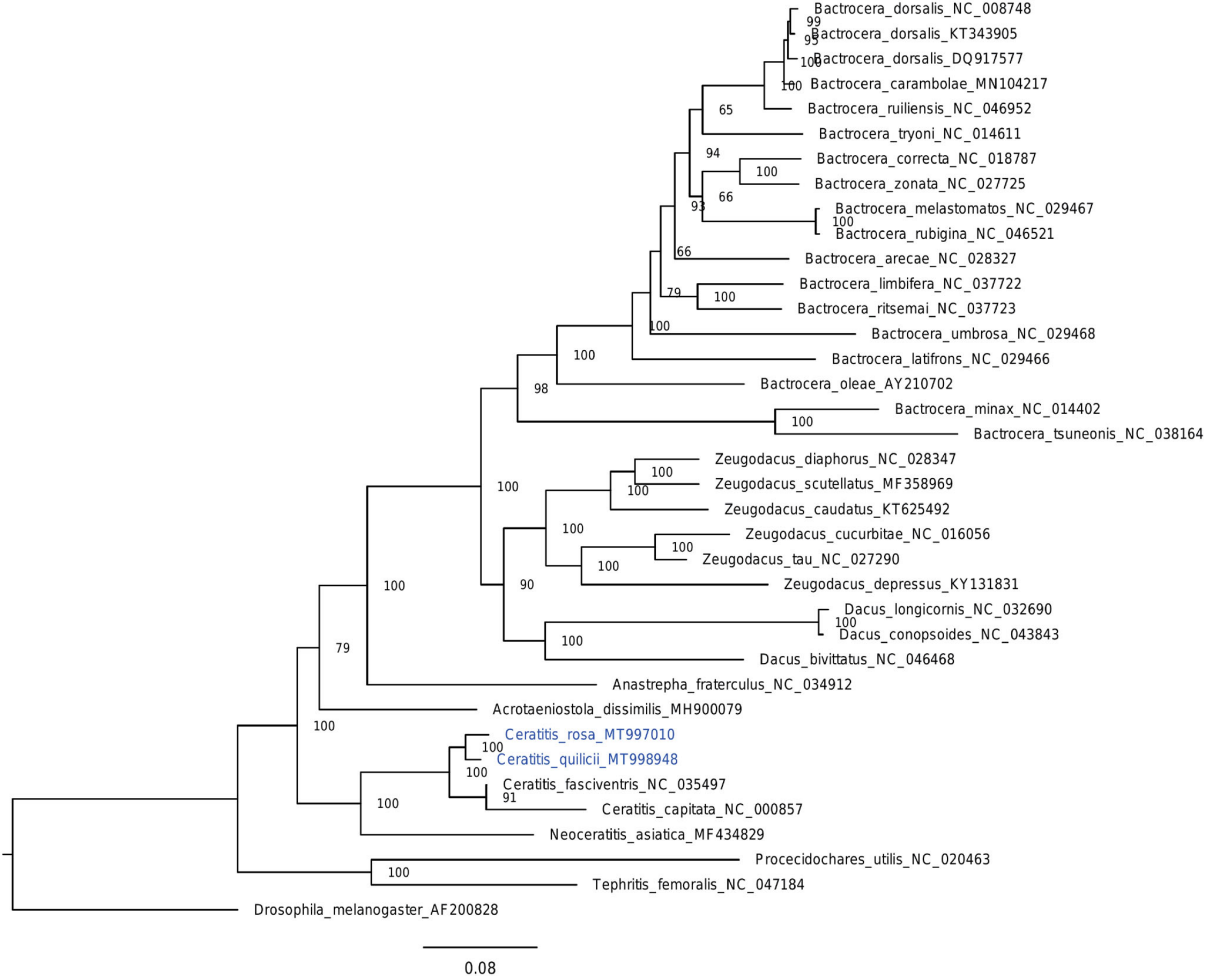
Phylogenetic tree based on the 13 PCGs of Tephritidae mitogenomes. Phylogeny was inferred in W-IQ-TREE. Numbers at nodes are for bootstrap percentages from 1000 replicates. Numbers following species names are GenBank accession numbers. The sequences analyzed in the present study are presented in blue.

## Data Availability

The genome sequence data that support the findings of this study are openly available in GenBank of NCBI at https://www.ncbi.nlm.nih.gov/ under the accession no. MT997010 (*C. rosa*) and MT998948 (*C. quilicii*). The associated BioProject, SRA, and Bio-Sample numbers are PRJNA680883, SRR13181787 and SRR13171161, and SAMN16925622 and SAMN16925623, respectively.
